# Novel approach for deriving genome wide SNP analysis data from archived blood spots

**DOI:** 10.1186/1756-0500-5-503

**Published:** 2012-09-13

**Authors:** Katie E Fowler, Chris P Reitter, Grant A Walling, Darren K Griffin

**Affiliations:** 1 School of Biosciences, University of Kent, Canterbury, Kent, CT2 7NZ, UK; 2 Department of Pathology, University of Cambridge, Tennis Court Road, Cambridge, CB2 1QP, UK; 3 JSR Genetics, Southburn, Driffield, East Yorkshire, YO25 9ED, UK

**Keywords:** FTA Whatman™^TM^ cards, Single nucleotide polymorphism, SNP, DNA extraction, Whole genome amplification, WGA, STR-PCR

## Abstract

**Background:**

The ability to transport and store DNA at room temperature in low volumes has the advantage of optimising cost, time and storage space. Blood spots on adapted filter papers are popular for this, with FTA (Flinders Technology Associates) Whatman™^TM^ technology being one of the most recent. Plant material, plasmids, viral particles, bacteria and animal blood have been stored and transported successfully using this technology, however the method of porcine DNA extraction from FTA Whatman™^TM^ cards is a relatively new approach, allowing nucleic acids to be ready for downstream applications such as PCR, whole genome amplification, sequencing and subsequent application to single nucleotide polymorphism microarrays has hitherto been under-explored.

**Findings:**

DNA was extracted from FTA Whatman™^TM^ cards (following adaptations of the manufacturer’s instructions), whole genome amplified and subsequently analysed to validate the integrity of the DNA for downstream SNP analysis. DNA was successfully extracted from 288/288 samples and amplified by WGA. Allele dropout post WGA, was observed in less than 2% of samples and there was no clear evidence of amplification bias nor contamination. Acceptable call rates on porcine SNP chips were also achieved using DNA extracted and amplified in this way.

**Conclusions:**

DNA extracted from FTA Whatman cards is of a high enough quality and quantity following whole genomic amplification to perform meaningful SNP chip studies.

## Findings

### Introduction

Extraction of DNA from white blood cells, although a routine procedure, can be time consuming and costly when large numbers of samples are to be isolated and stored. Indeed, full extraction and −20°C storage is sometimes unnecessary due to the fact that for many (e.g. PCR based) applications, only very small amounts are needed. For this reason, in recent years, many institutes and commercial organisations have taken to storing human or other animal DNA by means of dried blood spots on adapted filter paper. In this way, only a drop of blood is required and subsequent PCR based tests can be performed on the small amount of DNA isolated. Storage of DNA in this way is far more space and energy efficient [1].

A number of different approaches have been used to extract DNA from dried blood spots. DNA extracted using the proteinase K method has been used for the detection of perinatal HIV [2], the Chelex-100 method [3] and the TE method, which is Tris-EDTA (TE) buffer based extraction of DNA from dried blood in filter paper [4]. The Chelex-100 method has been reported to be as efficient as (or more so) than either the proteinase K method or a phenol-chloroform extraction.

FTA (Flinders Technology Associates) Whatman™^TM^ cards are a patented technology that can also be used to store DNA by means of dried blood spots. The cards are impregnated with a mixture of chemicals including free radical traps and strong protein denaturing buffers that hold the DNA on the card, stabilising and protecting it from damaging factors such as UV, oxidation and microbial degradation [5]. Many different types of sample can be stored on these cards including cultured cells, plant material, plasmids, bacteria, viral particles and also solid tissue. Blood was the sample type used in this study. Because of this, these cards are widely used in many fields including drug discovery, plasmid screening, molecular biology, animal identification, forensics, paternity testing, cancer screening, human genomic disease screening and neonatal screening [6]. Rajendram and colleagues described the successful extraction of bacterial DNA from FTA Whatman™^TM^ cards [7].

The manufacturers claim that DNA on the cards remains stable at room temperature for over 17 years, permitting compact storage and eliminating the need for freezing. They also claim that the separation of blood products that normally inhibit downstream applications such as PCR is not necessary. Indeed, the method of extraction from FTA Whatman™^TM^ cards is a relatively novel approach that allows nucleic acids to be ready for downstream applications such as PCR and sequencing. One drawback of storage and analysis by this approach however is the small quantity of DNA that is extracted.

The interrogation and analysis of both flat bed microarrays and single-nucleotide polymorphism (SNP) chips have a range of applications in scientific enquiry including clinical diagnostic studies [8], genome evolution studies, linkage disequilibrium mapping as well as genome wide association studies [9]. A sufficient quantity and quality of DNA is an essential prerequisite for a successful experiment and, typically, 50 ng/μl of high quality DNA is cited as a minimum required amount. Such a quantity is far more than is usually extracted from an FTA Whatman™^TM^ card and thus, if such archived material is to be used for SNP chip interrogation, a robust means of whole genome amplification (WGA) is necessary. In recent years it has been demonstrated the use of WGA on single or small numbers of cells and their application for array comparative genomic hybridisation (aCGH) and SNP chip analysis [10-12]. Here these investigations were extended to the use of small quantities of DNA extracted from FTA Whatman™^TM^ cards. In so doing, a novel approach through which meaningful whole genomic information can be derived from archived blood spots has been generated.

### Materials and methods

The Whatman™^TM^ ‘FTA classic’ card was the chosen platform for this study. This card has four sample areas for up to 500 μl of whole blood (one card per sample). A total of 288 porcine blood spots stored on these cards were sourced from JSR Genetics Ltd (Driffield, UK) from three breeds, Sire Line Large White (SLLW), Duroc and a synthetic Pietrain line (96 from each breed). The blood samples used in this study were originally taken as part of routine testing at JSR Genetics in order to determine the halothane status of the animals. FTA Whatman™^TM^ cards were spotted with blood at time of original testing.

The final method of DNA extraction from FTA Whatman™^TM^ cards is outlined below. Before this protocol was adopted as the optimum, the length of time at which the punches were heated to was tested (5, 10, 15, 20, 25 and 30 minutes) as well as the length of time that the punches were washed (vortexed for 5, 10 and 20 seconds). Using a 3 mm Harris Micro Punch and Harris cutting mat (Fitzco Inc, MN, USA), two punches were removed from one 11 mm single sample circle. These punches were then dispensed into 0.2 ml micro-centrifuge tubes and placed on a thermocycler (Eppendorf Mastercycler EP Gradient S) for 15 minutes at 80°C with no lid heating. The punches were then moved to 1.5 ml micro-centrifuge tubes using sterile forceps and the lids were left open and allowed to cool for 10 minutes. 1 ml of molecular grade water was then added to the sample and vortexed for 5 seconds before all the liquid was removed. This step was then repeated and 100 μl of sterile water was then added to each of the samples, which were placed in a thermocycler for 20 minutes at 96°C. After 20 minutes the tubes were flicked 4–5 times to resuspend the DNA; the punches were then removed from the solution after having squeezed out any excess water by gripping with forceps. The DNA was then suspended in the solution and was stored at −20°C until further use.

**Figure 1:**
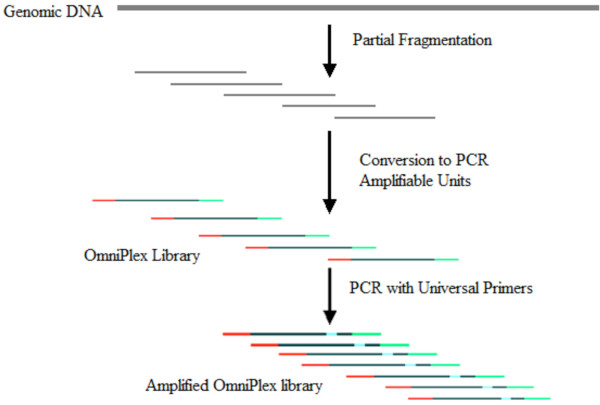
**Schematic of fragmentation based WGA from Sigma-Aldrich, sold under the name of WGA2.** Figure modified from www.sigma-aldrich.com.

Extracted samples were amplified via Whole Genome Amplification (WGA) using the Sigma-Aldrich WGA2 kit (see Figure 1), in order to produce an appropriate amount of DNA for microarray analysis. This fragmentation based WGA produces 400-600 bp overlapping fragments with defined 3’and 5’ ends and amplified via linear amplification followed by exponential amplification. This generates a 99% coverage of the genome. 10 μl of DNA from the above extraction was placed in a clean PCR tube and 1 μl of 10x fragmentation buffer was added. After centrifugation, tubes were heated to 95°C for exactly 4 minutes. Tubes were then placed on ice to cool and centrifuged before 2 μl of 1 x library preparation buffer and 1 μl of library stabilisation solution were added to each sample. After vortexing and centrifuging, samples were heated to 95°C for 2 minutes and then immediately cooled on ice. After centrifuging the samples, 1 μl of library preparation enzyme was added and then the tubes were placed in a thermocycler and incubated for 20 minutes at 16°C, 24°C, and 37°C respectively followed by 75°C for 5 minutes. After incubation, tubes were centrifuged before amplification. For the amplification steps, 7.5 μl of 10 x amplification master mix, 47.5 μl of nuclease-free water and 5 μl of WGA DNA polymerase was added to each of the samples, vortexed and centrifuged and placed in a thermal cycler for an initial denaturation period of 3 minutes at 95°C followed by 14 cycles of denaturation/extension of 15 seconds at 94°C and 5 minutes at 65°C. Samples were then either maintained at 4°C or stored at −20°C.

Short tandem repeats (STRs) are a type of molecular marker, which can be di-nucleotide (two bases repeating), tri-nucleotide (three bases repeating) or longer [13]. These short DNA sequences are repeated numerous times throughout the genome and can be different between individuals. PCR primers (developed in the early 1990s) which are designed to flank these regions are able to ‘fingerprint’ a genome [14]. Such primers are highly polymorphic, robust, easy to amplify and are also inexpensive. Two short tandem repeat primers (sourced from The Roslin Institute, Edinburgh, UK) were used for the purpose of amplifying the extracted DNA, SW268 (Forward: 5’-CTG ATT CAC TTT CAT TCG AGA A-3’, Reverse: 5’-AGC CCT TCC CTT AAT ATA ACC C-3’) and SW830 (Forward: 5’-AAG TAC CAT GGA GAG GGA AAT G-3’, Reverse: 5’-TGA GTG CAA CCG TGG TTA GG-3’). The reagents used for this PCR were 6.25 μl of PWO Master Mix (Roche, UK), 0.3125 μl of both forward and reverse primers, 1.625 μl of ddH_2_0 and 4 μl of genomic DNA. Samples were incubated for 2 minutes at 95°C, followed by 26 cycles of 95°C for 30 seconds, 61°C for 45 seconds and 72°C for 45 seconds, a final extension of 72°C for 10 minutes.

Fluorescent capillary electrophoresis is a method that separates ionic species by their size to charge ratio with fluorescent tags being used as the method for detection. 2 μl of Cy3 ladder was added to an aliquot of formamide (320 μl). Using a non-filter tip, 40 μl of this was pipetted into a 96-well opaque plate and 10-15 ng/μl of sample was added. One drop of mineral oil was added to the top of each of the wells. Buffer was added to a separate clear plate in the same locations as the samples in the opaque plate. Plates were subsequently loaded into a Beckman Coulter Genetic Analyser CEQ8000. One of four default separation methods, namely Frag-4 was implemented with an aim to employ the 600 set of standards (60 to 640 nucleotides). Frag-4 uses a higher separation temperature and a lower separation voltage, allowing for the allele patterns to be interpreted easily. Results were analysed by examination of the peaks produced and comparing results after PCR alone and also after WGA followed by PCR in order to ensure allele drop-out/amplification bias had not occurred.

Following the manufacturer’s instructions the Sigma GenElute PCR Clean-Up Kit was used to clean up whole genome amplified DNA. A GenElute Miniprep binding column was inserted into a 2 ml collection tube. 0.5 ml of column preparation solution was added to each column and centrifuged at 12000 g for 30 seconds and the eluate was subsequently discarded. 5 volumes of binding solution was added to 1 volume of amplified DNA and was added to the column. This was centrifuged at 16000 g for 1 minute and the eluate was once again discarded. 0.5 ml of wash solution was then added to the column and spun at 16000 g for 1 minute, discarding the eluate afterwards. Without any additional wash solution the column was spun for a further 2 minutes to remove excess ethanol. Any residual eluate and the collection tube were discarded and the column was transferred to a clean 2 ml collection tube. 50 μl of elution solution was applied directly to the centre of each column, incubated at room temperature for 1 minute and centrifuged at 16000 g for 1 minute. Products were stored at −20°C until needed. DNA quantification was performed as per section.

The Illumina Bead chip system (Illumina Porcine SNP60 Genotyping BeadChip) microarrays were interrogated with WGA amplified DNA from each porcine sample in an approach similar to that described by Handyside *et al.* 2010 [12] and Gabriel *et al.* 2011 [10], by colleagues at Cambridge Genomic Services, Department of Pathology, University of Cambridge. All DNA concentrations were adjusted to a concentration of 50 ng/μl, in a final volume of 5 μl per sample. The interrogation protocol was performed according to the manufacturer’s instructions (Illumina, Inc.). After WGA, the DNA was enzymatically fragmented to uniform sizes at 37°C for 1 hour, followed by 2-propanol precipitation and collection by centrifugation. DNA pellets were resuspended in a 150 μl total volume of hybridisation buffer, incubated at 48°C for 1 hour, and then heat denatured at 95°C for 20 minutes. A total of 83 μl for each sample was loaded onto each microarray and incubated at for 16 hours 48°C in a moist incubating chamber. After hybridisation, microarrays were washed to remove un-hybridised DNA template. Using a gravity flow heated *Tecan* manifold equilibrated at 44°C, all SNP chips underwent enzymatic single-base extension reaction, followed by two-colour immunofluorescence signal development, coated with a polymer resin and dried under vacuum for 1 hour to preserve signal fluorescence during scanning. The SNP chips were then scanned in the Illumina BeadStation 500 dual-laser, <1-micron resolution scanner, with laser excitations at 532 nm and 635 nm, emitting a spectra of 550–600 nm and 650–700 to score fluorescence values. Raw fluorescence data were captured and normalized, using internal and external controls, and stored as image files. Following scanning, image data were transferred to the GenomeStudio Software framework V2010.1 and converted from fluorescence data to genotypic data based on the manufacturer’s design algorithms. The ‘call rates’ produced by the Illumina software were determined. These call rates give an indication of the sample quality. A ‘call’ is defined as a genotype assignment while the call rate is the number of called SNPs (AA, AB or BB) divided by the total number of SNPs on the chip. A ‘No Call’ value occurs when a SNP’s probe intensity does not pass the detection filter score (DS) or the risk allele scores (RAS) fall outside of the statistical model boundaries [15].

### Results

A variation of the manufacturer’s recommendations was successfully developed to extract the DNA from FTA Whatman™ cards in order to give the optimum yield. Trial and error experimentation led to variations in the length of time that the punches from the card were heated to and the length of time that the punches were washed in molecular grade water. The optimum for which the punches were heated to was deemed to be 15 seconds (less or more than this led to less DNA being extracted) and the optimum length of time that the punches were washed for was deemed to be 5 seconds (more time had no impact on results).

Both the final protocol and the modifications are outlined above in the materials and methods. DNA was successfully extracted from 288/288 samples and amplified by WGA. The quantity and purity of DNA amplified was measured using a nanodrop spectrophotometer and using gel electrophoresis.

**Figure 2:**
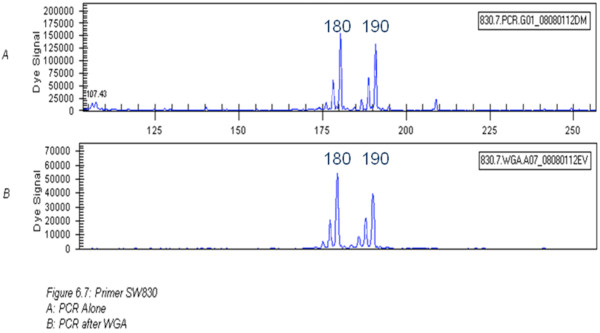
**An example of capillary electrophoresis results before (graph A) and after (graph B) WGA.** This confirms that no allele dropout or amplification bias occurred between the two steps as the peaks shown on the graphs are the same in both cases. In the case of amplification dropout, only one peak would be present in graph B.

Short Tandem Repeat Polymerase Chain Reaction (STR-PCR) established in all cases (apart from two), that WGA DNA was of the same origin as the directly extracted DNA (i.e. the WGA did not introduce any contamination). An example of the results from capillary electrophoresis is shown in Figure 2 and Table 1 shows an example of 20 samples (from the total 288) in which the DNA concentration was measured after WGA and the A260:A280 ratio was established. STR microsatellite analysis by fluorescent PCR results is also given. As this table shows, the concentrations of 20 tested samples after WGA were all above the 50 ng/μl required to be of sufficient quantity of SNP chip use. The purity (A260:A280) readings ranged from 1.80 to 2.14 (1.80 to 2.10 is generally considered to be in the acceptable range), with the exception of sample 9. This indicates failure of WGA in this sample with the results after fluorescent capillary electrophoresis being inconclusive. Another sample (18) showed evidence of allele drop out with only one of the two alleles amplifying. These samples were not taken further for SNP chip analysis. A total of less than 2% of the total sample size showed evidence of allele drop-out. The slightly different peak heights (from the STR-PCR profiles) represent different amplification efficiencies on the two alleles. This is very common in such experiments, especially when using WGA amplified material.

**Table 1:** Concentrations and A260/A280 for 20 samples after WGA (before amplification, concentrations too low for detection)

**Sample**	**Allele size(s) after extraction and microsatellite PCR**	**Concentration (ng/μl) after WGA**	**A260:A280 after WGA**	**Allele size(s) after WGA and microsatellite PCR**	**Do direct extraction and WGA microsatellite results match?**
1	119	612	2.10	119	✓
2	182	561	2.06	182	✓
3	119, 142	555	1.88	119, 142	✓
4	120	609	2.14	121	✓
5	119, 139	587	1.84	119, 139	✓
6	119, 149	580	1.80	119, 149	✓
7	120, 140	521	1.98	120, 140	✓
8	119, 137	534	1.97	119, 137	✓
9	121, 147	575	1.62	No peaks	X – WGA failure
10	118, 144	600	1.85	118, 144	✓
11	119	451	2.01	120	✓
12	119, 148	603	2.03	119, 148	✓
13	117	601	2.14	118	✓
14	120, 140	742	1.98	120, 141	✓
15	119, 142	598	1.86	120, 142	✓
16	118, 140	561	1.87	118, 140	✓
17	119, 137	604	2.05	119, 137	✓
18	121, 144	624	2.14	144	X – Allele drop out
19	142	612	2.07	141	✓
20	119, 146	709	1.95	119, 146	✓

According to the manufacturer’s claims, the DNA on the cards remains stable for up to 17 years [16]. Although this time frame was beyond the scope of this research, original experiments were repeated three years later (using the same samples as used in the original study) in order to verify that the DNA from the cards could still be extracted and amplified after storage at room temperature. The results displayed in Table 2 (showing an example of ten samples) indicate that the concentrations and purity of the extracted DNA (both before WGA and after) were comparable with no significant differences between the means and standard deviations (p = 0.05). The coefficient of variation for these ten samples (expressed as a percentage) is also shown; this is a normalised measure of dispersion.

**Table 2:** Concentrations and A26/A280 measurements for 10 samples initially and three years after the first experiment

**Sample**	**Up to one year after sampling**		**3-4 years after sampling**	
	**Concn. (ng/μl) after WGA**	**A260:A280 after WGA**	**Concn. (ng/μl) after WGA**	**A260:A280**
1	612	2.10	632	1.97
2	561	2.06	704	2.01
3	555	1.88	745	1.91
4	609	2.14	784	2.07
5	587	1.84	672	1.85
6	580	1.80	691	1.94
7	521	1.98	587	1.98
8	534	1.97	521	2.08
9	575	1.62	612	1.85
10	600	1.85	528	2.12
Mean	573.4	1.92	647.6	0.98
SD	30.71	0.16	87.7	0.09
C_v_ (%)	5.35	8.19	13.55	4.74

The call rates (percentage success rate of experiment) obtained when genomic DNA was used on the porcine Illumina SNP chip were typically between 0.80 and 0.96, with an average of 0.91 and a standard deviation of 0.046. Table 3 provides a list of call rates for 96 samples. These were, on the whole lower than we had observed with DNA from human samples however entirely consistent with other genomic DNA experiments on these SNP chips (Cambridge Genomic Services, personal communication). In order to determine whether there was a bias in genome coverage caused by the WGA protocol, SNP chip data were re-analysed and specific regions of the genome that consistently failed to amplify were noted (see Table 4). Consistent failure was considered to have occurred if there was no call for 100 animals or more.

**Table 3:** Call rates produced from the Illumina Porcine SNP60 Genotyping BeadChip for 96 Duroc samples

**Sample ID**	**Call Rate**	**Sample ID**	**Call Rate**	**Sample ID**	**Call Rate**
DF1	0.9398113	DF33	0.8864012	DL18	0.9231989
DF2	0.9049336	DF34	0.8883713	DL19	0.7598297
DF3	0.89525	DF35	0.8968361	DL20	0.7989148
DF4	0.9002421	DF36	0.9486268	DL21	0.9390433
DF5	0.9039319	DF37	0.9508974	DL22	0.9180065
DF6	0.9047333	DF38	0.9324318	DL23	0.9143167
DF7	0.8911929	DF39	0.9125637	DL24	0.931263
DF8	0.8725603	DF40	0.9184573	DL25	0.9369397
DF9	0.896235	DF41	0.9150513	DL26	0.9087069
DF10	0.898055	DF42	0.9167376	DL27	0.8873028
DF11	0.9493948	DF43	0.917105	DL28	0.8955839
DF12	0.9276066	DF44	0.5607814	DL29	0.8741297
DF13	0.9290425	DF45	0.9409466	DL30	0.9085566
DF14	0.925336	DF46	0.951081	DL31	0.8999249
DF15	0.9223976	DF47	0.9278404	DL32	0.8899741
DF16	0.9197429	DF48	0.9347191	DL33	0.8869021
DF17	0.9151515	DL1	0.9212956	DL34	0.9033642
DF18	0.9077553	DL2	0.9166207	DL35	0.9114784
DF19	0.9292262	DL3	0.9031973	DL36	0.9263545
DF20	0.9323316	DL5	0.9003757	DL37	0.9229819
DF21	0.9417815	DL6	0.9007764	DL38	0.9225144
DF22	0.9224977	DL7	0.8644461	DL39	0.9228817
DF23	0.9103431	DL8	0.8700225	DL40	0.9225311
DF24	0.917873	DL9	0.9578429	DL41	0.9174055
DF25	0.9250021	DL10	0.9476584	DL42	0.9159863
DF26	0.9200768	DL11	0.9445696	DL43	0.8470657
DF27	0.9235997	DL12	0.9464062	DL44	0.9348026
DF28	0.8814926	DL13	0.92759	DL45	0.9609317
DF29	0.8847149	DL14	0.929126	DL46	0.9478754
DF30	0.9086067	DL15	0.9290258	DL48	0.9106603
DF31	0.8877035	DL16	0.912163	DL49	0.9345021
DF32	0.8846481	DL17	0.8998581	DL50	0.9391435

**Table 4:** Table shows regions of the genome considered biased after whole genome amplification

**Chromosome**	**Start Position**	**End Position**	**Number of Animals**
1	9,948,603	10,128,760	137
1	18,418,830	18,641,391	115
2	4,278	9,124,477	163
2	1,632,514	3,559,307	106
2	73,598,562	106,082,295	125
4	120,957,396	128,105,906	129
7	48,160	11,045,685	135
7	58,390,175	64,149,907	135
9	27,950	7,940,294	118
11	52,003	6,263,717	141
11	60,971,377	68,843,993	179
14	137,688,899	148,678,088	185
16	46,422,064	58,631,201	165

SNP chip data were re-analysed and specific regions of the genome that consistently failed to amplify were noted. Consistent failure was considered to have occurred if there was no call for 100 animals or more.

The results therefore demonstrate the effectiveness of FTA Whatman™^TM^ cards as a means of porcine genomic DNA storage for subsequent SNP chip analysis.

### Discussion

This study has successfully demonstrated the efficacy of FTA Whatman™^TM^ cards as a tool for storing porcine genomic DNA at room temperature largely supporting the manufacturer’s claims. Moreover, it demonstrates the use of prior WGA and effective interrogation of the Illumina SNP60 Genotyping BeadChip. The reported call rates were comparable to those commonly seen in our own experience and very similar to published data when non-amplified porcine DNA was used [17-19].

For the first time, it can clearly be seen that genomic DNA can be extracted from FTA Whatman™^TM^ cards after three years post initial blood spotting. Due to the large sample number in this study, storage of whole blood is inconvenient at −20°C due to limited freezer space. When using FTA Whatman™^TM^ cards however the samples were able to be collected at the same time and processed before each experiment.

A similar DNA extraction study was carried out in 2002 in birds which produced comparable results to those obtained in this study [6]. In this case, FTA Whatman™^TM^ cards were also evaluated for their use of long-term blood storage as well as for sex-determination via PCR. A similar study was carried out in birds with respect to avian influenza. In this case, FTA Whatman™^TM^ cards as a means of ensuring biosafety [20] and mentions the problems virology studies face with regard to preservation of samples, as well transportation and maintenance (solved by FTA Whatman™^TM^ cards). Another comparable study was carried out in pigs as a way of detecting porcine reproductive and respiratory syndrome [21]. Once again, the issue of sample acquisition and means of sample storage was mentioned in this paper and stated the benefits of FTA Whatman™^TM^ cards as a means of storage.

A study in 2011 [22] compared three different sources of DNA for GWAS using forensic human samples (degraded genomic DNA (both amplified and unamplified) and amplified DNA from FTA Whatman cards). This study also showed that DNA extracted from FTA cards with subsequent amplification was suitable for GWA studies however, STR PCR was not performed to confirm that no allele drop out or amplification bias had occurred. Our study has also indicated that porcine DNA is stable within the card’s matrix at least 3 years.

### Conclusions

FTA Whatman™^TM^ cards have been shown to be very useful for the storage of genomic porcine (and presumably other species’) DNA at room temperature and extraction of DNA from these cards has been shown to be stable for at least 3 years. The extracted DNA was shown to be of a high enough concentration and quality (after WGA) to perform meaningful SNP chip studies. This could, in turn, facilitate many other studies for which sample acquisition and storage is problematic and facilitate other studies with reference to both SNPs (e.g. genome wide association studies) and copy number variants (CNVs) amongst others.

## Abbreviations

FTA: Flinders Technology Associates; DNA: Deoxyribonucleic acid; SNP: Single nucleotide polymorphism; WGA: Whole Genome Amplification; PCR: Polymerase chain reaction; aCGH: array Comparative Genomic Hybridisation; SLLW: Sire Line Large White; DS: Detection filter Score; RAS: Risk Allele Score; STR: Short tandem repeat.

## Competing interests

All authors declare that they have no conflict of interest.

## Authors’ contributions

KF performed most of the experiments in the paper and co-wrote the manuscript, CR performed the microarray experiments and generated the call rate data, GW co-conceived the project and collected all the samples and DG co-conceived the project, supervised all aspects of it and co-wrote the manuscript. All authors read and approved the final manuscript.
